# The Changing Values of English Speech

**Published:** 1909-05

**Authors:** 


**The Changing Values of English Speech.** By **Ralcy Husted Bell.** New York: Hinds, Noble & Eldridge, Publishers.

This book is designed as a companion work to the Worth of Words, and it is not only valuable as a contribution, but it is exceptionally well written in Dr. Bell's invigorating style even if —and this is said in all sincerity—it be myopic in one place.

The author very properly condemns scientific writings as being done into exceeding poor English, and declares the pulpit to be fairly free from common errors of speech. It is to the editorial writers of the newspapers that he gives credit as the greatest and strongest conservators of our language "because their influence for good form is constant and far reaching." None will disagree with him in this; it is quite true; but he falls into with disregard of facts when he says: "The average 'reporter' is not particularly a paragon in many and nameless essentials; and yet, be it said to his eternal glory, his English is far from vile when one considers the kind of stuff he drinks and the stress and action laid upon his work."

It is quite likely that in his search for examples the author has been led into the narrow by-ways of hasty and yellow journalism. The daily newspaper, it must be admitted, does frequently contain glaring errors of grammatic construction; but the average reporter, as a rule, is a writer of pure English. He may at times drop into the vernacular, and it is doubtful if even Dr. Bell would hesitate to admit that even here one may find gems of speech. He does the "average reporter" an injustice. There are in every large city an hundred "average reporters" who write English of such virgin purity that it would seem to properly belong within the pages of a school girl's book. It is certain that the "average reporter" will be glad to learn that his English is not "absolutely vile" in the opinion of the author.

The telling of the development of language is excellent, well done and historic. In the chapter devoted to Intensitives the author gives us many delightful and material pages. He also brings in that exquisite story about Henry W. Grady's visit to a Boston home of culture and refinement where, when the guests were departing, the hostess said: "Mr. Grady, do say something original. All my guests have said they have had a delightful time or something equally trite and stupid. I expect something better of you." And Grady, in his courtliest manner gave it to her: "Madam, I have had one hell of a time." Did the lady get fussed? Not a bit. She wasn't even surprised; but in tones of perfect breeding she answered: "Mr. Grady, I am damned glad of it."

There is little in the book to criticise. It is one of the volumes—one of the very few—which one really enjoys and from which one gets nothing but good. The chapter on English orthography and simplified spelling tells all about the various and abortive attempts to make spelling easy, from the very first effort to the present day display of activities of the self-constituted board which starting with a dozen, and has finally become mired in a maze of mysterious and lop-eared words. If one has an honest yearning for solid information he'll get it in this book and plenty of it.

